# Alcohol and Cancer Stem Cells

**DOI:** 10.3390/cancers9110158

**Published:** 2017-11-20

**Authors:** Mei Xu, Jia Luo

**Affiliations:** Department of Pharmacology and Nutritional Sciences, University of Kentucky College of Medicine, 1095 Veterans Drive, Lexington, KY 40536, USA; mxu222@uky.edu

**Keywords:** alcoholism, carcinogenesis, HER2/ErbB2, metastasis, stemness

## Abstract

Heavy alcohol consumption has been associated with increased risk of several cancers, including cancer of the colon, rectum, female breast, oral cavity, pharynx, larynx, liver, and esophagus. It appears that alcohol exposure not only promotes carcinogenesis but also enhances the progression and aggressiveness of existing cancers. The molecular mechanisms underlying alcohol tumor promotion, however, remain unclear. Cancer stem cells (CSC), a subpopulation of cancer cells with self-renewal and differentiation capacity, play an important role in tumor initiation, progression, metastasis, recurrence, and therapy resistance. The recent research evidence suggests that alcohol increases the CSC population in cancers, which may underlie alcohol-induced tumor promotion. This review discusses the recent progress in the research of alcohol promotion of CSC and underlying cellular/molecular mechanisms. The review will further explore the therapeutic potential of CSC inhibition in treating alcohol-induced tumor promotion.

## 1. Introduction

Alcohol abuse is a major public health concern. Ethanol, as the major constituent of alcoholic beverages, and its metabolite acetaldehyde were classified as carcinogenic to humans [1]. It is now well established that alcohol consumption is a risk factor for human cancer. It is estimated that 3.6% of all cancers worldwide (1.7% in women, 5.2% in men) are attributable to alcohol consumption [2]. Alcohol abuse also attributed 3.2% to 3.7% of cancer deaths in the United States [3]. While alcohol exposure may enhance the carcinogenesis or initiation of cancers, it may as well increase the aggressiveness and malignancy of existing tumors. However, the underlying mechanisms remain elusive. A better understanding of these mechanisms is critical in developing effective therapeutic strategies for cancer patients who drink alcoholic beverages. 

Experimental studies clearly demonstrate that alcohol alters the behavior of cancer cells and transforms them into more aggressive phenotypes [4]. For example, in breast cancer cells, alcohol increased mobility and invasive potential; it also promoted the epithelial-mesenchymal transition (EMT), a hallmark of malignancy, and impaired endothelial integrity, thereby increasing the dissemination of breast cancer cells and facilitating metastasis. Alcohol also stimulated tumor angiogenesis through the activation of cytokines and chemokines, which promotes tumor growth. Recent research progress indicates that alcohol may target cancer stem cells (CSCs), a subpopulation of cancer cells with self-renewal and differentiation capacity. We will first review the evidence on alcohol’s effects on CSCs, and then discuss the potential underlying cellular and molecular mechanisms.

## 2. Alcohol Alters CSC Population

Many cancer patients develop tumor recurrence or metastasis, and resistance to therapy. Research of tumor biology has led to the hypothesis that tumors may possess a stem cell-like subpopulation known as CSCs that drive tumor propagation and pathogenesis. CSCs are small subpopulations of cells within tumors, and may arise from normal stem cells or progenitor cells following transforming mutations and result from epigenetic plasticity as well as interconversion and dedifferentiation of non-CSCs to CSCs [5,6]. CSCs are identified and characterized by the expression of distinctive cell surface and intracellular markers, and can be differentially separated from non-CSCs [7]. The tumors generated from CSCs express the phenotypic heterogeneity of the parent tumor containing mixed populations of CSCs and non-CSCs. CSCs are responsible for all of the important characteristics of tumors, including tumor initiation, heterogeneity, therapy resistance, recurrence, and metastasis [7].

We have recently shown that alcohol increased the mammary CSC population both in vitro and in vivo models [8,9]. Using aldehyde dehydrogenase (ALDH) activity as an intracellular marker and CD44^+^/CD24^−/low^ as cell surface markers [10], we were able to identify and quantify CSC population within breast cancer cells. We showed that alcohol exposure (100 mg/dL) for 10 days caused an increase in the CSC population in cultured MCF-7 breast cancer cells and MCF-7 cells overexpressing ErbB2 (MCF-7-ErbB2 cells). The increased CSC population was accompanied by the formation of mammospheres, an increase in cell migration/invasion, anchorage-independent colony formation, and scattering spheroids in a 3-D Matrigel system [8,9]. Interestingly, alcohol-induced increase in CSC population in MCF-7-ErbB2 cells was much more than MCF-7 cells which expressed low ErbB2 levels. The findings were confirmed by animal studies which showed that chronic alcohol (12 months) exposure increased CD44 positive cells in the mammary tumors of MMTV-neu transgenic mice which was accompanied by the increased metastases in the lung and colon [9].

Alcohol-induced CSCs was also observed in liver cancer [11,12]. Alcohol feeding induced CD133^+^/CD49f^+^ liver CSCs. Nanog is one of the core transcription factors found in pluripotent embryonic stem cells and an important marker/regulator of CSCs; Toll-like receptor 4 (TLR4) is upstream of Nanog and involved in the malignant transformation of liver cancer cells. Alcohol activated TLR4-Nanog pathway may underlie alcohol-induced liver CSCs [13]. Using a mouse model of alcohol-driven hepatocellular carcinoma (HCC), Ambade et al evaluated (2016) the effect of alcoholic steatohepatitis on early hepatobiliary carcinoma after initiation by diethyl-nitrosamine (DEN) [12]. Mice-treated by Alcohol + DEN showed hepatobiliary cysts and early hepatic neoplasia. Proliferation markers (BrdU, cyclin D1, p53) and CSC markers (CD133 and Nanog) were significantly up-regulated in the livers of alcohol-fed and DEN-injected mice compared to controls. 

Alcohol consumption increases the risk of developing cancer of the oral cavity, pharynx and esophagus [14]. It is proposed that alcohol may enhance these cancers by promoting the divisions of the stem cells that maintain tissue in homeostasis. Alcohol could cause a local cytotoxic effect on the cells lining the epithelial tissues of the oral cavity, pharynx, and esophagus. In response to alcohol cytotoxicity, the stem cells located in deeper layers of the mucosa, replicated to replace the dead cells. The enhanced division of stem cells made them prone to carcinogen-mediated mutations, resulting in malignant transformation [14]. In a mouse model of carcinogenesis of head and neck squamous cell carcinoma (HNSCC), alcohol and carcinogen 4-nitroquinoline-1-oxide (4-NQO) worked together to promote carcinogenesis [15]. 4-NQO plus alcohol exposure resulted in massive, horizontal expansion of stem/progenitor cell populations arising from single stem cells in the basal layer of the epithelia. This expansion was consistent with carcinogen-associated, symmetric division of stem/progenitor cells. 

It appears that alcohol also affects non-CSCs. In an attempt to understand the effect of alcohol on stem cells, Khalid et al. (2014) investigated the alcohol-induced alterations in genes and DNA methylomic changes in human embryonic stem cells (hESCs). Alcohol caused significant alterations in gene profiles of hESCs, particularly those associated with molecular pathways for metabolic processes and oxidative stress. A genome-wide DNA methylome analysis revealed that alcohol induced widespread alterations in the methylation of many regions of chromosomes in the hESCs [16]. Dental pulp stem cells (DPSCs), also known as dental pulp-derived mesenchymal stem cells, are a multipotent adult stem cell population that has high proliferative potential. Similar to the findings in hESCs, alcohol affected a significant number of genes in DPSCs by altering their DNA methylation [17].

## 3. Cellular and Molecular Mechanisms Underlying Alcohol Stimulation of CSCs 

### 3.1. Oxidative Stress and Tumor Microenvironment in Alcohol Promotion of CSCs 

Reactive oxygen species (ROS) accumulation and oxidative stress have been proposed as important mechanisms for carcinogenesis and cancer aggressiveness [4]. ROS is known to regulate CSC property [6,18]. Being exposed to environmental stressors, including ROS, CSCs may adapt and develop antioxidant systems to improve ROS defense capability and acquire a malignant phenotype [6]. Although oxidative stress has been extensively associated with apoptosis in cancer cells, it may regulate survival signaling depending on its levels. For example, ROS generation is linked to the stimulation of the pro-survival pathway which is driven by PI3K/AKT and cytokine signaling [6]. It is likely that ROS-adaptive responses may stimulate survival signaling pathways in CSCs and maintain CSC characteristics.

Alcohol exposure produced ROS in both tumor cells and stromal cells [4]. Alcohol-induced ROS and oxidative stress may be mediated by alcohol metabolism, damaged mitochondria, and an antioxidant response [19]. Alcohol is first oxidized to acetaldehyde in the cytosol by alcohol dehydrogenase (ADH). Acetaldehyde is transported into the mitochondria and rapidly metabolized to acetate by aldehyde dehydrogenase 2 (ALDH2). In the mitochondria, acetate is converted to acetyl-CoA, which enters the citric acid cycle for ultimate oxidization. In both ADH and ALDH2 catalyzed reactions, NAD^+^, is used as an electron carrier to form NADH, which is eventually transported into the mitochondria for ATP production. Depending on oxygen supply and the demand of ATP, NADH may not be efficiently oxidized, which causes electrons to be diverted to form ROS, causing oxidative stress. During chronic alcohol consumption or in tissues that lack ADH, cytochrome P450 2E1 (CYP2E1) is induced to engage in alcohol metabolism, which concomitantly oxidizes NADPH to generate ROS. Alternatively, alcohol can activate NADPH oxidase (NOX) which produces ROS [20]. 

CSCs reside in niches which are pockets of distinct microenvironments with specific functional characteristics. Such pockets are regulated by a variety of factors and cell types, including immune cells, cancer-associated fibroblasts, extracellular matrix (ECM) components, cytokines/chemokines, angiogenesis, hypoxia, and pH [5]. Taken together, alcohol-induced ROS may affect the microenvironment in CSC niches, impacting their fate and properties, such as differentiation, survival, and self-renewal ability. For example, alcohol has been shown to alter ECM components, MMP activity, stromal cell function, cytokines/chemokines, hypoxia-induced factors, etc., which change the CSC microenvironment and then regulate CSC fate and property [12,21,22,23,24,25,26,27]. 

### 3.2. Epidermal Growth Factor Receptor (EGFR) Family in Alcohol Promotion of CSCs

The EGFR family is comprised of four structurally similar receptors including E) and ErbB4 (HER4) [28,29]. They are type I trans-membrane kinase receptors, which upon ligand binding in the extracellular domain, undergo dimerization and subsequent trans-phosphorylation in the intracellular domain. EGFR and ErbB2 receive particular attention in the context of breast cancer etiology and therapy because of their frequent overexpression and hyperactivation in breast carcinomas [28]. Overexpression of EGFR and ErbB2 is associated with malignant breast cancers, increased metastasis, and poor prognosis [29,30,31,32,33]. Both EGFR and ErbB2 play an important role in regulating cancer stemness [34,35,36].

Alcohol stimulated the phosphorylation of EGFR and regulated EGFR signaling in mammary epithelial cells and breast cancer cells [37,38]. Alcohol exposure caused a drastic increase in the GFR (ErbB1 or HER1), ErbB2 (HER2), ErbB3 (HER3 CSC population and mammosphere formation in breast cancer cells overexpressing ErbB2, but it has a modest effect on breast cancer cells expressing low levels of ErbB2 [8,9]. Consistently, alcohol exposure increased the CSC population and induced ErbB2 phosphorylation in the mammary tumors of MMTV-neu mice. Both in vitro and in vivo studies indicate that alcohol significantly increased the phosphorylation of ErbB2 in breast cancer cells and mammary epithelial cells expressing high levels of ErbB2, but has little effect on cells with low levels of ErbB2 [9,24,39]. Therefore, it is possible alcohol regulates cancer stemness by activating EGFR and ErbB2.

### 3.3. p38γ MAPK in Alcohol-Induced Increase in CSC Population

There are four p38 MAPK isoforms, p38α, p38β, p38γ and p38δ. p38γ is particularly implicated in breast cancer progression and aggressiveness [40]. p38γ activation promoted the development and progression of triple-negative breast cancer (TNBC) by stimulating CSC expansion. p38γ silencing in TNBC cells reduced mammosphere formation and decreased expression levels of CSC drivers including Nanog, Oct3/4, and Sox2 [41]. We showed that alcohol selectively activated p38γ but no other isoforms in an ErbB2-dependent manner [8]. Inhibition of ErbB2 abolished alcohol-induced activation of p38γ and its interaction with the substrate, SAP97/DLG. Moreover, the activation of ErbB2/p38γ/SAP97/DLG axis appeared to mediate alcohol-induced increase CSC population as well as migration/invasion; blocking p38γ significantly inhibited alcohol-stimulated increases of CSC population and mammosphere formation [8]. In a mouse model HNSCC, alcohol-induced activation of p38 MAPK and β-catenin was accompanied by a significant expansion of stem/progenitor cell populations arising from single stem cells in the basal layer of the epithelia, suggesting the involvement of p38 MAPK and β-catenin [15].

RhoC, a member of Rho family of GTPase, is a downstream effector of p38γ, and has been suggested to enhance cell mobility and metastasis through the degradation and reconstruction of the ECM and induction of angiogenic factors [42,43,44]. Recent studies indicated that RhoC is an important regulator of CSCs [45]. The alcohol-induced activation of p38γ upregulates RhoC levels by promoting RhoC stability which resulted in enhanced migration/invasion [8]. Increased levels of RhoC may impact CSC population following alcohol exposure. 

### 3.4. Other Signaling Proteins Regulating Stemness in Alcohol Promotion of CSCs

Wnt/GSK3β/β-catenin signaling pathway plays an important role in tumorigenesis, tumor progression and cancer therapy [46]. Recent studies showed that Wnt/GSK3β/β-catenin pathway was involved in the regulation of CSCs [47,48]. We showed that alcohol activated Wnt/GSK3β/β-catenin pathway in colon cancer cells, resulting in an increase in the migration/invasion of colon cancer cells [49]. Chronic alcohol exposure stimulated the Wnt/GSK3β/β-catenin signaling pathway in the liver, which increased hepatocyte proliferation thus promoting tumorigenesis [50]. Nanog and OCT4 are well-established CSC transcription factors responsible for the stemness network [51]. Alcohol exposure (25 mM) for 4 weeks increased the expression of Nanog and OCT4 in MCF-7 breast cancer cells [52]. In a mouse model of liver carcinogenesis, alcohol activated TLR4-Nanog pathway, which was accompanied by the induction CSCs [13]. 

## 4. Summary, Future Studies, and Potential Therapeutic Approaches

**Figure 1:**
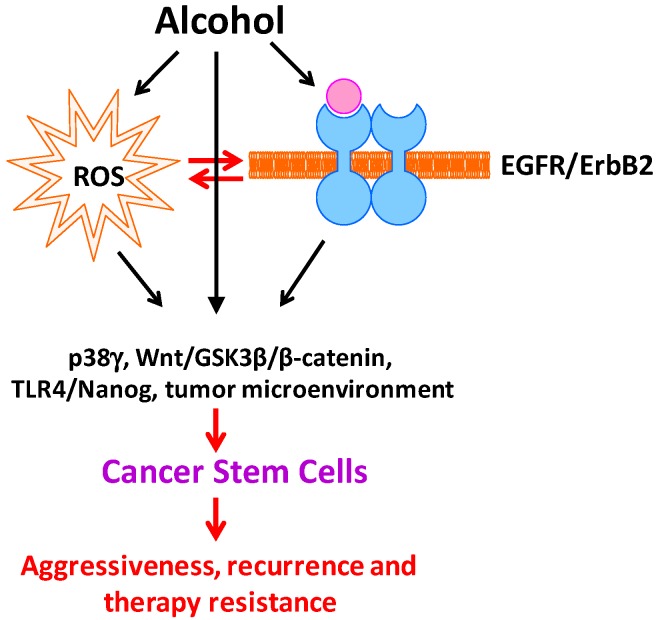
Effect of alcohol on cancer stem cells. Alcohol exposure induces oxidative stress and the activation of EGFR/ErbB2. There is a considerable interaction between ROS and EGFR/ErbB2 activation in response to alcohol exposure. Alcohol-induced ROS and EGFR/ErbB2 activation triggers signaling cascades and alters microenvironments that are responsible for stemness, differentiation, proliferation and survival of CSCs, therefore increasing CSC population. Increased CSCs convey aggressive, recurrence and therapy resistance.

Available evidence indicates that alcohol increases CSC population, and therefore may promote aggressiveness, recurrence, and therapy resistance of cancers. Alcohol may initially activate EGFR/ErbB2 and/or induce ROS, which stimulate critical signaling components, such as p38 MAPK, Wnt/GSK3β/β-catenin, and TLR4/Nanog, as well as alter tumor microenvironment, resulting in the promotion of CSCs (Figure 1). In addition to the signaling pathways discussed above, several signaling cascades also regulate the fate and properties of CSCs, such as, JAK/STAT, Hedgehog, Notch, TGF-β, and HIPPO-YAP/TAZ [6,7]. Future studies will need to carefully evaluate these signaling pathways in the context of alcohol-induced promotion of CSCs. So far, studies have been focusing on the action of alcohol. The role of acetaldehyde, a product of alcohol metabolism, is unclear. Acetaldehyde are mutagens which can form adducts with proteins and DNA, inducing gene mutation, DNA crosslinks and chromosomal aberrations and are involved in carcinogenesis. The effect of acetaldehyde on CSCs is worthy of investigation. Since alcohol affects multi-components/cascades involved in tumor progression and aggressiveness, therapeutic approaches may need to simultaneously target multiple components or cascades, which will likely yield more effective therapeutic outcomes particularly for alcoholic cancer patients. In addition to conventional therapeutic efforts, targeting stemness pathways and CSC niches may offer more effective treatments for alcoholic cancer patients. The use of novel approaches, such as small-molecule inhibitors of specific proteins in signaling pathways that regulate stemness, proliferation and migration of CSCs, or immunotherapy and noncoding microRNAs targeting CSCs, may provide better means of treating malignant cancers as well as cancer progression associated with alcohol exposure. For example, several drugs targeting CSC differentiation and cell death pathways are in clinical trial, [53,54]. More importantly, owing to the evidence that alcohol increases breast cancer progression, primary care givers should question breast cancer patients about their alcohol intake. If the consumption is determined excessive, the patient should be informed about its consequences and be recommended that alcohol intake be prohibited or reduced.

## Abbreviations

ADH	alcohol dehydrogenase
ALDH2	aldehyde dehydrogenase
CSC	cancer stem cells
CYP2E1	cytochrome P450 2E1
ECM	extracellular matrix
EGFR	epidermal growth factor receptor
HCC	hepatocellular carcinoma
HNSCC	head and neck squamous cell carcinoma
MMP	matrix metalloproteinase
ROS	reactive oxygen species
